# Influence of Preoperative Cervical Spine Curvature in Postoperative Radiographic Outcomes Following Anterior Cervical Discectomy and Fusion: A Single-Center Retrospective Study

**DOI:** 10.7759/cureus.89497

**Published:** 2025-08-06

**Authors:** Ashwin Ghadiyaram, Asha Krishnakumar, Andrew K Schwieder, Thérèse F Weidenkopf, Hayden M Dux, Jefferson O Abaricia, Charles F Opalak, Robert S Graham, William C Broaddus

**Affiliations:** 1 Department of Surgery, State University of New York Upstate Medical University, Syracuse, USA; 2 Department of Surgery, University of Washington, Seattle, USA; 3 Department of Surgery, Eastern Virginia Medical School, Norfolk, USA; 4 Department of Neurosurgery, Virginia Commonwealth University, Richmond, USA; 5 Department of Neurological Surgery, Oregon Health & Science University, Portland, USA; 6 Department of Neurosurgery, Southeastern Neurosurgical and Spine Institute, Greenville, USA

**Keywords:** anterior cervical discectomy and fusion (acdf), cervical alignment, cervical lordosis, kyphosis, radiographic, spine surgery

## Abstract

Background

Anterior cervical discectomy and fusion (ACDF) is a common surgical procedure that patients undergo for cervical disc herniations and degenerative disc disease, aimed at relieving radicular symptoms and restoring cervical alignment. The impact of preoperative kyphotic cervical imbalance versus preoperative lordosis on postoperative radiographic outcomes in ACDF patients is unclear. The purpose of this study is to examine how preoperative cervical sagittal balance can influence quantified postoperative cervical sagittal balance.

Methods

This retrospective study studied patients who had an ACDF at the Virginia Commonwealth University Medical Center from 2005 to 2017. Patients with preoperative and postoperative cervical radiographs, with radiographs of at least 1.5 years postoperatively, were included. While follow-up time frames varied from patient to patient, participants were excluded if they had any missing preoperative or postoperative imaging at least 1.5 years after the surgery date. Additionally, patients were excluded if they had prior cervical spine surgery or trauma, or if they had missing information on postoperative or preoperative imaging. Cervical sagittal parameters, including the atlantoaxial C1-C2 angle, C2-C6 Cobb angle, C7 slope, and cervical sagittal vertical axis (cSVA), were measured using Surgimap, an analytical radiographic imaging software. Patients were divided into preoperative lordotic and kyphotic cohorts based on preoperative C2-C6 Cobb angle.

Results

Of the 507 patients undergoing ACDF, 65 met the inclusion criteria. There was no significant difference in follow-up between the surgery date and the first postoperative follow-up, among the kyphotic (129.8 ± 296.2 days) and lordotic (142.7 ± 393.3 days) cohorts (p=0.88). There was also no significant difference between the cohorts in the total follow-up duration from the time of surgery to the last follow-up (kyphotic:1217.2±803.7 days; lordotic: 1640.8±1112.7 days; p=0.087). The kyphotic cohort had a significant change in the C2-C6 Cobb angle postoperatively (-10.0º (95% CI -14.197º-5.809º); p<0.0001), sustained through the last documented follow-up (net change: -8.956º (95% CI -12.952º-4.960º), p<0.0001). There was no significant difference in the Cobb angle in the lordotic cohort both at the first postoperative follow-up (-2.19º (95% CI 6.76º -2.37º); p=0.34) and at the last documented follow-up (net change: 2.03º (95% CI 3.20-7.25); p=0.44). The postoperative Cobb angles at the last documented follow-up in the kyphotic (-2.6º±10.9º) and lordotic (-6.3º±13.1º) cohorts did not vary significantly (p=0.23).

Conclusion

Long-term improvements following ACDF in patients with preoperative kyphosis suggests that the procedure, in the long term, is effective in restoring structural cervical lordosis and radiographic properties in the maintenance of cervical sagittal alignment, regardless of preoperative cervical alignment. Future studies can assess how preoperative kyphosis and lordosis influences clinical outcomes, such as functionality and pain following ACDF to determine whether preoperative alignment has a role in the procedure's outcomes.

## Introduction

Anterior cervical discectomy and fusion (ACDF) is the most common procedure for cervical disc herniations and cervical degenerative disc disease, with approximately 132,000 procedures performed every year [1,2]. ACDF is primarily indicated for cervical nerve root decompression in patients with radiculopathy, supported by Class I evidence of its efficacy in treating cervical radicular symptoms [3,4]. It is a commonly chosen procedure for cervical realignment procedures due to the level of patient satisfaction, short hospitalization, and proven radiological fusion [5].

According to the North American Spine Society 2013 Appropriate Use Criteria, anterior fusion surgery is indicated in cases of radiculopathy, regardless of cervical sagittal balance before surgery [6]. The role of cervical sagittal balance following ACDF surgery has been increasingly studied in recent years to determine whether cervical lordotic function is significantly impacted by fusing two or more vertebrae, thereby changing the load-bearing forces within the cervical vertebral column [7,8].

Through cage or graft implantation, cervical retraction, and the use of anterior internal fixation with a plate and screws in ACDF procedures, restoration of the cervical spine, and significant improvement in height and anatomic curvature can often be achieved. Along with kyphotic alignment correction, there is an increased change in cervical sagittal vertical axis (cSVA) post-ACDF [9,10]. The ratio of cervical lordosis to cervical slope (CL/C7s), which is correlated with increased global spine alignment, is used to estimate cSVA [11]. Furthermore, cSVA is an important postoperative outcome that has been increasingly recognized in the literature as associated with improved quality of life [12].

A metric commonly used in studying cervical misalignment, aside from cSVA and CL/C7s, is the T1 slope minus the cervical lordosis (TS-CL) [13]. TS-CL has been used as a measurement for moderate or severe disability [14] and has been shown to be significantly correlated with the distance between the C2 to C7 plumb line, a vertical line drawn down the vertebral bodies of both vertebrae [14]. The C7 slope (C7s) has been increasingly used over TS because of the higher radiographic visibility of C7 over T1 [15]. Thus, C7s minus cervical lordosis (C7s-CL) can be an indicator of cervical sagittal imbalance [15,16].

The atlantoaxial C1-C2 angle is also used in examining cervical imbalance. Studies have shown that the C1-C2 angle correlates with the C2-C7 Cobb angle [17], a common measure of cervical sagittal alignment and one of the most frequently used measures of cervical angular alignment for determining kyphosis and lordosis in the cervical spine [18]. A positive Cobb angle (>0º) indicates lordosis, while a negative Cobb angle (<0º) is indicative of kyphosis [19]. As the lower cervical vertebrae may be hard to visualize on a radiograph, a validated acceptable surrogate for a C2-C7 Cobb angle is the C2-C6 Cobb angle [20].

Restoration of optimal lordosis has been studied as an outcome measure representing success in spinal surgery [21]. In cervical spinal surgery, loss of cervical lordosis (LCL) has been studied as a negative outcome measure to detect problems encountered in cervical laminoplasty [22]. CL restoration globally realigns spinal segments, whereas malalignment of the cervical spine can negatively impact alignment of the thoracolumbar angle and pelvis [23].

The influence of preoperative cervical alignment on cervical alignment outcomes following ACDF has been unclear [24]. The purpose of this study is to examine how preoperative kyphosis or lordosis may influence the quantified radiographic outcomes of cervical alignment following ACDF. A part of this article was previously presented as a meeting abstract at the 2021 American Association of Neurological Surgeons (AANS) Annual Scientific Meeting on August 23, 2021.

## Materials and methods

Patient selection

This retrospective study included patients who underwent an ACDF with the Department of Neurological Surgery at the Virginia Commonwealth University (VCU) Medical Center between 2005 and 2017. Patients were identified using a list of Current Procedural Terminology (CPT) codes and were screened for inclusion based on their history and available imaging data. While surgical technique consisted of graft implantation in all patients with an autograft or synthetic bone graft, the technique was not specifically controlled for in the analysis. Patients who had upright, neutral cervical images before, after, and greater than 1.5 years after surgery were included. Exclusion criteria consisted of patients who had prior cervical spine surgery or trauma. All patients included had measurements taken throughout the follow-up period. No patients lost to follow-up were included in the study. The requirement for informed consent was waived because of its retrospective and de-identified nature, and this study, calling for retrospective chart review, was approved by the Virginia Commonwealth University Institutional Review Board (approval no. HM20007990).

Cervical alignment measurements

**Figure 1:**
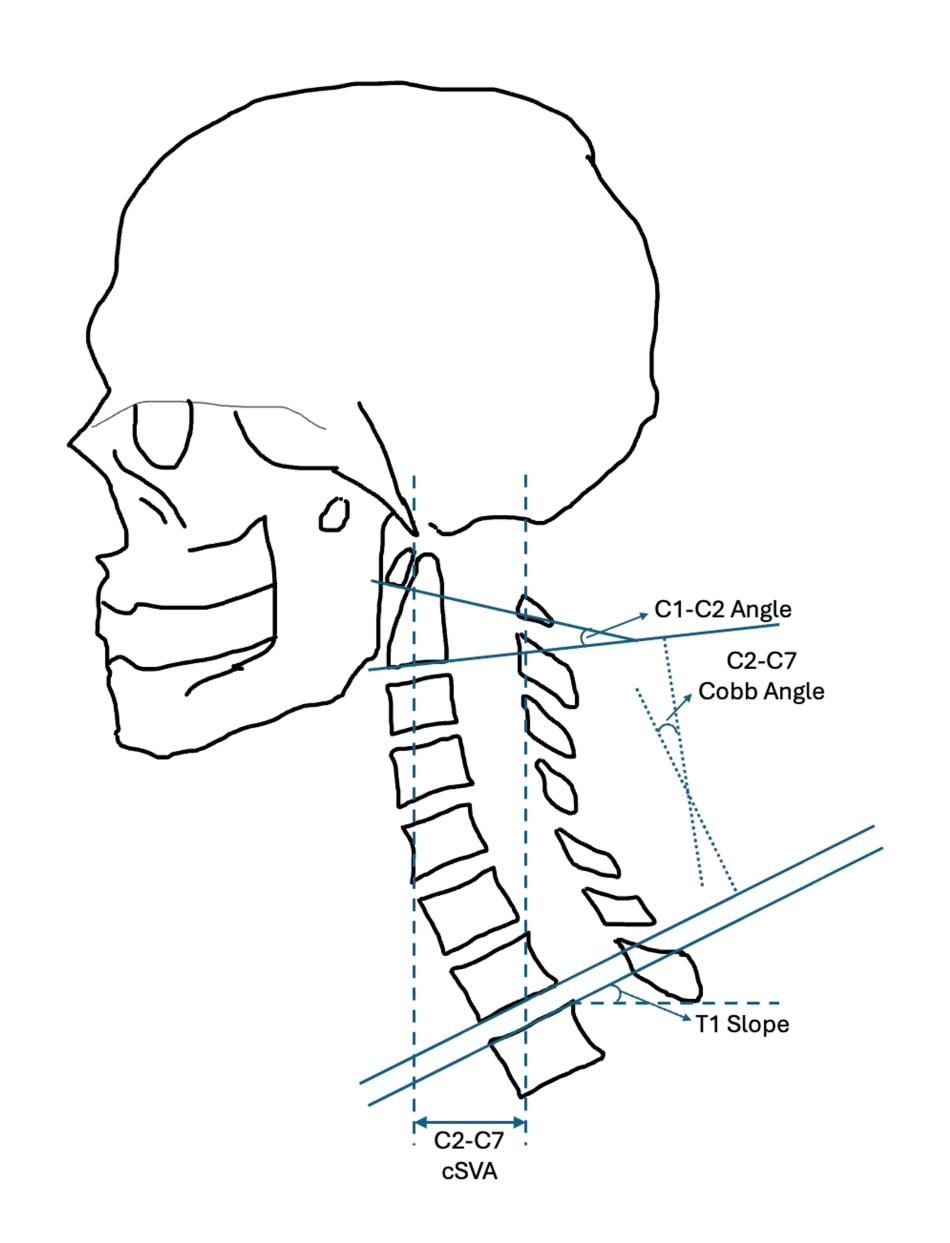
Measurements used to assess cervical sagittal alignment Image created by Dr. Krishnakumar using Powerpoint (Microsoft Corp., Redmond, WA, US).

The preoperative and postoperative images of patients included in the study were scanned into analytical radiographic imaging software and measured using the Cervical Wizard program. The measurements were verified by multiple researchers to increase interrater reliability and measurements were also confirmed with multiple neurosurgeons to preserve accuracy. The analytical radiographic imaging software, Surgimap® (Nemaris Inc.; Methuen, MA, USA) is a program developed as a tool for viewing, organizing, and measuring radiographic images. The program has developed a series of software tools for measuring radiographic images of the spine. The Cervical Wizard is a program for measuring cervical angles by superimposing measurements onto cervical X-rays, CT scans, and MRIs. By feeding the program the locations of the superior endplate of C7, the inferior endplate of C2, and the angle of C1, the software can measure the C2-C6 Cobb angle, C1-C2 angle, C2-C7 cSVA, and the C7 slope. The measurements taken by the Cervical Wizard are illustrated in Figure 1. 

**Figure 2:**
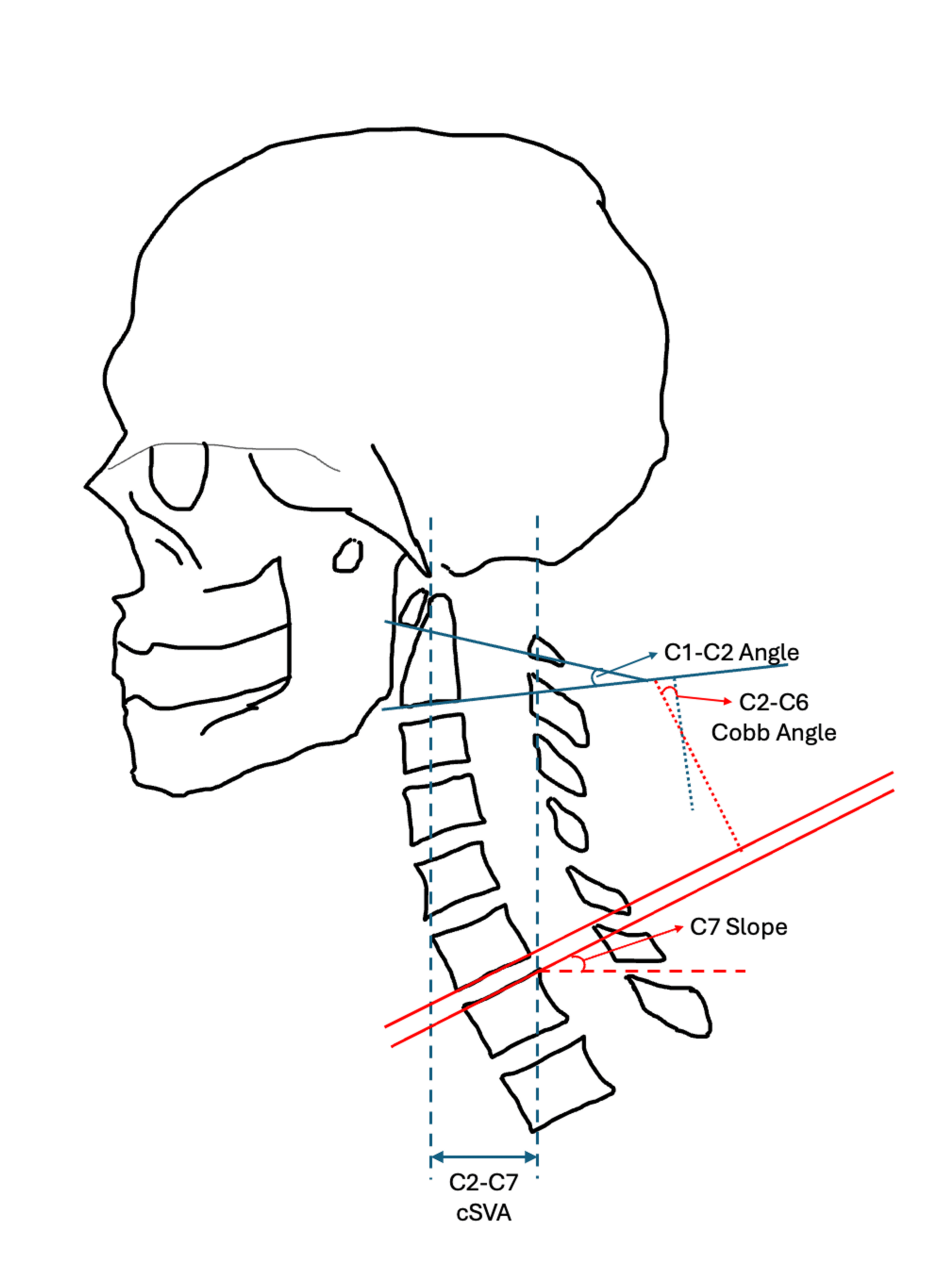
Measurements used in this study to assess cervical sagittal alignment in patients who underwent ACDF, with the modified measurements in red ACDF: Anterior Cervical Discectomy Fusion; Image created by Dr. Krishnakumar using Powerpoint (Microsoft Corp., Redmond, WA, US).

Due to the limitations in several images, many radiographs in this study did not have clear views of the superior surface of the T1 vertebra. Instead, the superior surface of the C7 vertebra was used, and to reduce bias and increase consistency of measurements, inferior measurements reported by the Cervical Wizard were shifted up one level, resulting in the Cobb angle being read from C2-C6, and the C7 slope being used in place of the T1 slope. The shift of measurements has been validated in the literature for providing an accurate measure of the C2-C7 Cobb angle and the T1 slope [20]. The Cobb angle was used as the CL measure for calculating C7s-CL. As a result, the measures collected with analytical radiographic imaging software included the C2-C6 Cobb angle, C7s, C7s-CL, and the C2-C7 cSVA, as illustrated in Figure 2. 

Stratification of patient cohorts

**Figure 3:**
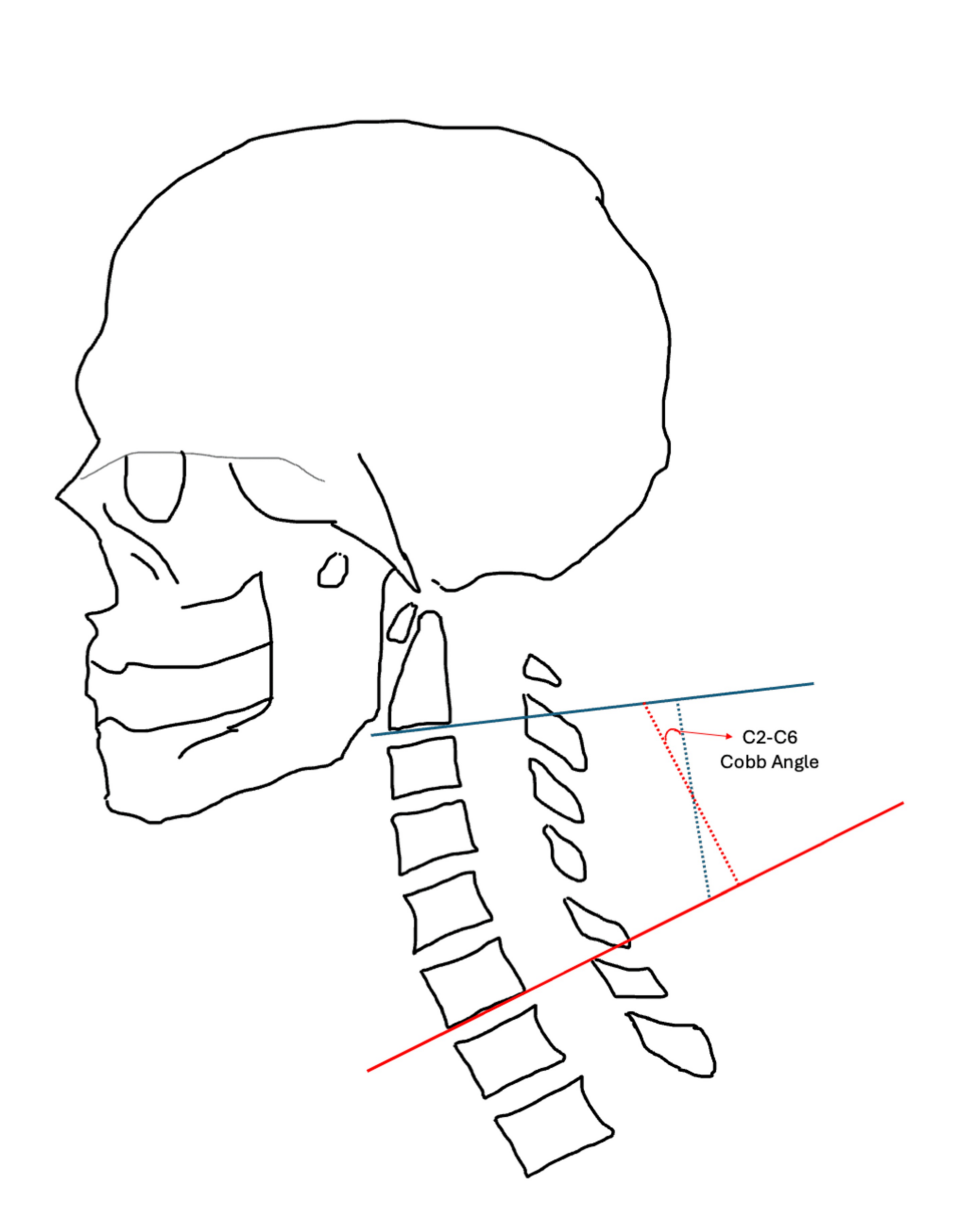
Sagittal alignment of cervical lordosis, and how to measure the C2-C6 Cobb angle Image created by Dr. Krishnakumar using Powerpoint (Microsoft Corp., Redmond, WA, US).

**Figure 4:**
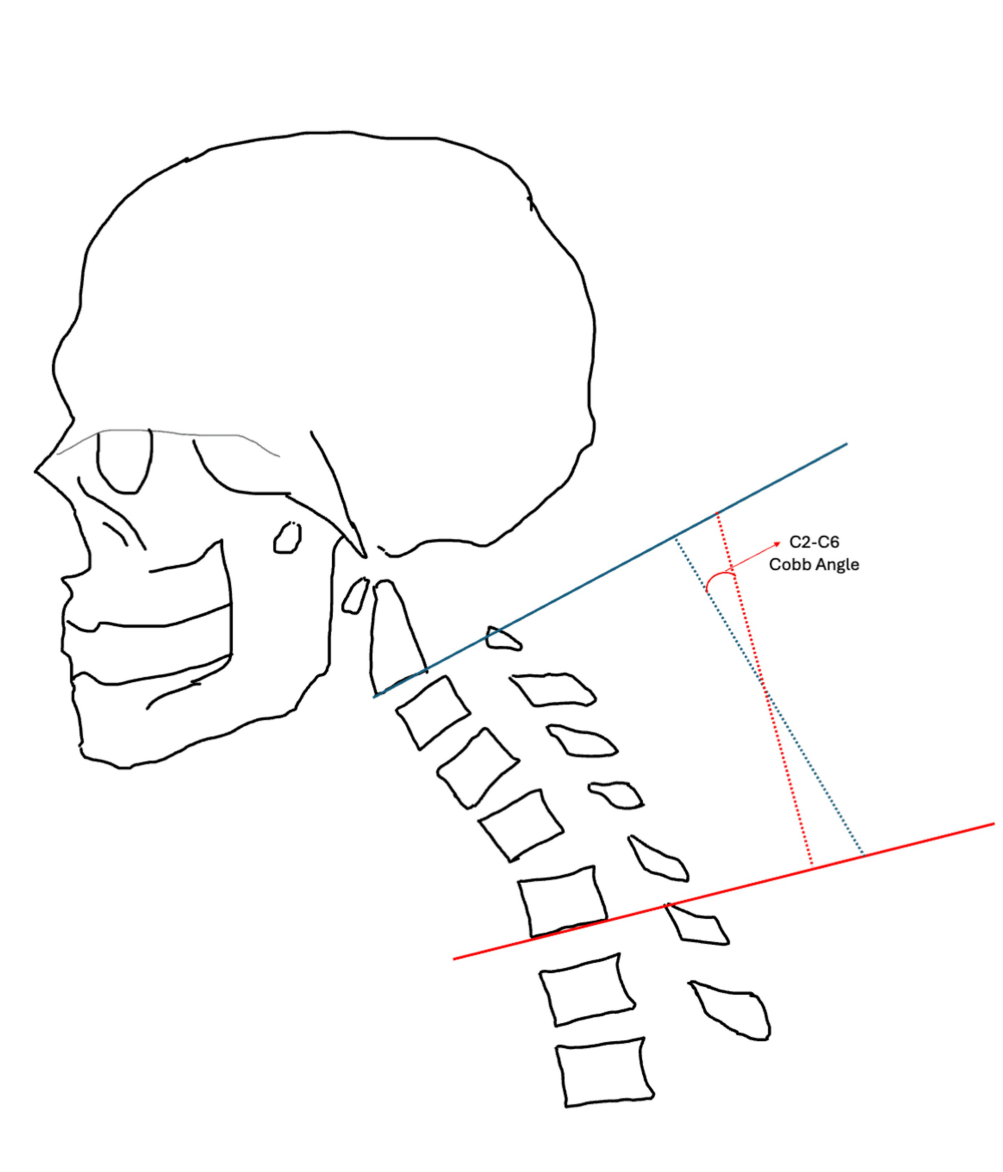
Sagittal alignment of cervical kyphosis, and how to measure the C2-C6 Cobb angle Image created by Dr. Krishnakumar using Powerpoint (Microsoft Corp., Redmond, WA, US).

Patients were grouped into pre-operative lordotic or kyphotic groups based on their C2-C6 Cobb angle measurement before surgery. Cobb angles were measured by drawing two horizontal lines, one at the inferior endplate of C2 and one at the inferior endplate of C6. A perpendicular line was drawn to each horizontal line and the angle at the intersection of both perpendicular lines was where the Cobb angle was measured [18-19]. Cobb angle measurements for both a lordotic cervical spine and kyphotic cervical spine are illustrated in Figures 3, 4, respectively. Patients with a negative Cobb angle were considered lordotic while those with a positive Cobb angle were considered kyphotic.

Statistical analysis

Descriptive statistics, including demographics and clinical and surgical characteristics were performed using chi-squared tests for categorical variables and t-tests for continuous variables. The Cervical Wizard provided measurements of the C1-C2 angle, C2-C6 Cobb angle, the cSVA, and C7s, and a calculated C7s-CL. Using these measurements, the sagittal balance in the preoperative images of patients who had undergone ACDF were compared to the image from the first postoperative follow-up and the last documented postoperative follow-up. Measurements were noted as mean ± SD and differences in measurements were noted as mean differences with a 95% confidence interval. Comparisons between preoperative and postoperative images were performed within each cohort with paired t-tests and between cohorts using unpaired t-tests.

Measured differences in each measurement were compared at the last documented follow-up; the two differences being compared were between the measurements at preoperative and the first postoperative visit, and between the measurements at first preoperative and the last documented postoperative visit. The differences within cohorts were compared using paired t-tests, and between cohorts were compared using unpaired t-tests.

For each comparative t-test assessing radiographic outcomes, a Cohen’s d value was calculated to determine the effect size of the comparison and finding [25]. Cohen’s d values of 0.2, 0.5, and 0.8 were minimum benchmarks of a small, medium, and large effect size, respectively [25]. Values below 0.2 were interpreted as a negligible effect size [25].

## Results

In the present study, charts of 507 patients were reviewed, and 65 patients met the inclusion criteria. Baseline patient characteristics are summarized in Table 1.

**Table 1:** Demographic characteristics of the patients in the study These patients underwent Anterior Cervical Discectomy and Fusion (ACDF) Surgery at Virginia Commonwealth University from 2005-2017

Characteristics	Kyphotic (n=34)	Lordotic (n=31)	p-value
Gender, n (%)			1.00
Male	17 (50.0)	15 (48.4)	
Female	17 (50.0)	16 (51.6)	
Race, n (%)			0.089
Black	19 (55.9)	9 (29.0)	
White	15 (44.1)	22 (71.0)	
Smoking status, n (%)			0.63
Current smoker	14 (41.2)	10 (32.3)	
Former/Non-smoker	20 (58.8)	20 (64.5)	
Unknown	0 (0)	1 (3.2)	
Age, years (mean ± SD)	48.23 ± 10.0	53.0 ± 10.0	0.060
Number of levels fused, n (%)			0.98
2	14 (41.2)	12 (38.7)	
3	11 (32.4)	10 (32.3)	
4	8 (23.5)	7 (22.6)	
5	1 (2.9)	2 (6.5)	
Follow-up (mean ± SD)			
Days from initial visit to surgery	99.2 ± 139.3	125.4 ± 245.5	0.60
Days from surgery to first follow-up	129.8 ± 296.2	142.7 ± 393.3	0.88
Days from first follow-up to last documented follow-up	988.2 ± 727.2	1247.3 ± 1156.8	0.29
Days from surgery to last documented follow-up	1118.0 ± 795.8	1515.4 ± 1137.8	0.11
Days from initial visit to last documented follow-up	1217.2 ± 803.7	1640.8 ± 1112.7	0.087

Out of the included patients, 34 (52.3%) were in the kyphotic cohort, while 31 (47.7%) were in the lordotic cohort. The gender distribution was similar in both cohorts, with the kyphotic cohort having 17 (50%) and the lordotic cohort having 15 (48.4%) male patients (p=1.00). There were no significant differences between the cohorts in terms of racial distribution, with the kyphotic cohort comprising 44% White participants and 56% Black participants, and the lordotic cohort being made up of 71% White patients and 29% Black patients (p=0.09). In the kyphotic cohort, 14 patients (41.2%) were current smokers and 20 patients (58.8%) were former smokers, while in the lordotic cohort, 10 patients (32.3%) were current smokers and 20 patients (64.5%) were former smokers (p=0.63). The mean age (± SD) was 48.2±10.0 years in the kyphotic cohort and 53.0±10.0 years in the lordotic cohort (p=0.06).

In the kyphotic cohort, 14 (41.2%) patients underwent a two-level ACDF, 11 (32.4%) underwent a three-level ACDF, eight (23.5%) underwent a four-level ACDF, and one (2.9%) underwent a five-level ACDF. In the lordotic cohort, 12 (38.7%) patients underwent a two-level ACDF, 10 (32.3%) underwent a three-level ACDF, seven (22.6%) underwent a four-level ACDF, and two (6.5%) underwent a five-level ACDF. The distribution of patients undergoing ACDF, by the number of levels fused, had no significant difference (p=0.98). The average follow-up duration (±SD) from the initial visit to the last postoperative visit was 1217.2±804 days in the kyphotic cohort and 1640.8±1113 days in the lordotic cohort and the between-cohort difference was not significant (p=0.087). The mean time interval (±SD) from surgery to the first postoperative visit was 129.8±296.2 days in the kyphotic cohort and 142±393.3 days in the lordotic cohort (p=0.88). The time from the first preoperative follow-up and the last documented postoperative follow-up was 988.2±727.2 days in the kyphotic cohort and 1247.3±1156.8 days in the lordotic cohort (p=0.29). Lastly, the mean time interval from the surgery to the last documented follow-up was 1118.0±795.8 days in the kyphotic cohort and 1515.4±1137 days in the lordotic cohort (p=0.11).

Information on the preoperative cervical spinal measurements are summarized in Table 2.

**Table 2:** Preoperative cervical spine measurements (mean ± SD)

	Kyphotic cohort (n=34)	Lordotic cohort (n=31)	p-value	Cohen’s d
C2-C7 cSVA (mm)	16.9 ± 6.3	15.5 ± 7.2	0.40	0.21
C1-C2 angle (degrees)	29.1 ± 6.0	29.2 ± 5.7	0.93	0.021
C2-C6 angle (degrees)	6.4 ± 4.3	-8.3 ± 6.3	<0.0001	2.75
C7s (degrees)	15.9 ± 6.2	23.5 ± 6.0	<0.0001	1.22
C7-CL (degrees)	22.3 ± 5.7	15.2 ± 7.7	<0.0001	1.06

These include cSVA, C1-C2 angle, C2-C6 Cobb angle, C7s, and C7s-CL. Preoperative comparisons calculated with unpaired t-tests between the two cohorts are also summarized in Table 2. Preoperative C2-C6 Cobb angles were significantly higher in the kyphotic cohort, by virtue of being positive, with a negative average angle in the lordotic cohort (p<0.0001; d=2.75). C7s was significantly greater in the lordotic cohort than in the kyphotic cohort (p<0.0001; d=1.22). On the other hand, C7-CL was greater in the kyphotic than in the lordotic cohort (p<0.0001; d=1.06). There were no significant differences between the cohorts in preoperative cSVA and preoperative C1-C2 angle.

Postoperative measurements in both cohorts at the first postoperative follow-up and the last documented follow-up visit, along with unpaired t-test comparisons, are summarized in Table 3. 

**Table 3:** Postoperative cervical spine measurements (mean ± SD) cSVA: cervical sagittal vertical axis; CL: cervical lordosis; C7s: cervical slope

	First postoperative follow-up				Last documented postoperative follow-up			
	Kyphotic cohort (n=34)	Lordotic cohort (n=31)	p-value	Cohen’s d	Kyphotic cohort (n=34)	Lordotic cohort (n=31)	p-value	Cohen’s d
C2-C7 cSVA (mm)	22.1 ± 8.8	24.1 ± 9.5	0.38	0.22	20.6 ± 9.4	20.7 ± 11.9	0.96	0.014
C1-C2 angle (degrees)	30.0 ± 7.0	28.6 ± 7.5	0.43	0.20	30.5 ± 6.1	28.0 ± 12.2	0.30	0.27
C2-C6 angle (degrees)	-3.6 ± 11.5	-10.5 ± 11.0	0.017*	0.21	-2.6 ± 10.9	-6.3 ± 13.1	0.23	0.31
C7s (degrees)	23.3 ± 8.3	28.6 ± 7.8	0.01*	0.66	22.7 ± 8.3	23.8 ± 15.0	0.72	0.091
C7-CL (degrees)	19.6 ± 8.4	18.1 ± 8.5	0.47	0.18	20.1 ± 8.4	17.5 ± 10.7	0.28	0.27

At the first postoperative follow-up, the kyphotic cohort had a significantly less negative angle than the lordotic group (p=0.017; d=0.21), whereas the lordotic group had a significantly greater C7s than the kyphotic group (p=0.01; d=0.66). There were no significant differences in cSVA, C1-C2 angle, and C7-CL between the cohorts at the first postoperative follow-up. Moreover, there were no significant differences in the C2-C6 Cobb angle and C7s between the cohorts at the last documented postoperative follow-up. Other measurements, such as the cSVA, C1-C2 angle and C7-CL also did not vary significantly between the cohorts at the last documented postoperative follow-up (Table 3).

The difference in cervical alignment measurements between the preoperative and the first postoperative measurements for both cohorts are summarized in Table 4.

**Table 4:** Differences between the pre- and postoperative cervical spine measurements between the cohorts at the first postoperative follow-up, along with between-cohort comparison of the change Mean diff: Postoperative measurement minus preoperative measurement; CI: Confidence Interval; cSVA: cervical sagittal vertical axis; CL: cervical lordosis; C7s: cervical slope

	Kyphotic cohort (n=34)			Lordotic cohort (n=31)			Comparison of the change between the cohorts	
	Mean diff (95% CI)	p-value	Cohen’s d	Mean diff (95% CI)	p-value	Cohen’s d	p-value	Cohen’s d
C2-7 cSVA (mm)	5.15 (1.45–8.86)	0.007	0.67	8.59 (4.29–12.88)	0.064	1.02	0.12	0.39
C1-C2 angle (degrees)	0.94 (-2.21–4.08)	0.55	0.14	-0.63 (-4.04–2.78)	0.57	0.09	0.30	0.26
C2-C6 angle (degrees)	-10.0 (-14.20–5.81)	<0.0001	1.16	-2.19 (-6.76–2.37)	0.34	0.24	0.0018	0.81
C7s (degrees)	7.31 (3.76–10.86)	0.0001	1.00	5.10 (1.54–8.66)	0.006	0.73	0.24	0.30
C7s-CL (degrees)	-2.69 (-6.15–0.77)	0.13	0.38	2.89 (1.22–7.01)	0.16	0.36	0.0058	0.72

In the kyphotic cohort, the C2-C7 cSVA (5.2 mm; p=0.007; d=0.67) and C7s (7.3º, p=0.0001; d=1.00) were significantly increased and the C2-C6 Cobb angle (-10.0º; p<0.0001; d=1.16) was significantly decreased by the first postoperative visit after ACDF. On the other hand, there were no significant postoperative changes in C1-C2 angle and the C7-CL in this cohort. In the lordotic cohort, the C7s increased (5.10º; p=0.006; d=0.73) postoperatively. However, there were no postoperative changes in cSVA, C1-C2 angle, C2-C6 Cobb angle, and C7-CL.

Comparisons of the difference in each measurement between the preoperative and the first postoperative measurement among the cohorts are also given in Table 4. After the first postoperative visit, there was a significant difference between the kyphotic and lordotic cohorts in the changes in C2-C6 Cobb angle (p=0.0018; d=0.81) and the C7-CL (p=0.0058; d=0.72). Conversely, there was no significant between-cohort differences in the cSVA, C1-C2 angle, or C7s at the first postoperative follow-up.

Differences in the cervical alignment measurement between the preoperative and the last documented follow-up were compared and summarized for both cohorts in Table 5.

**Table 5:** Differences between pre- and postoperative cervical spine measurements in both cohorts at the last documented follow-up, along with between-cohort comparison of the change Mean diff: Postoperative measurement minus preoperative measurement; CI: Confidence Interval; cSVA: cervical sagittal vertical axis; CL: cervical lordosis; C7s: cervical slope

	Kyphotic (n=34)			Lordotic (n=31)			Comparison of the change between the cohorts	
	Mean diff (95% CI)	p-value	Cohen’s d	Mean diff (95% CI)	p-value	Cohen’s d	p-value	Cohen’s d
C2-7 cSVA (mm)	3.65 (-0.23–7.53)	0.065	0.46	5.23 (-0.22–10.23)	0.041	0.53	0.63	0.12
C1-C2 angle (degrees)	1.40 (-1.51–4.31)	0.34	0.24	-1.25 (-6.07–3.58)	0.61	0.13	0.28	0.28
C2-C6 angle (degrees)	-8.96 (-12.95–4.96)	<0.0001	1.09	2.03 (-3.20–7.25)	0.44	0.20	0.001	0.88
C7s (degrees)	6.76 (3.21–10.31)	0.0003	0.92	0.32 (-5.49–6.12)	0.91	0.028	0.061	0.48
C7s-CL (degrees)	-2.18 (-5.65–1.29)	0.22	0.30	2.33 (-2.41–7.07)	0.33	0.25	0.087	0.44

In the kyphotic cohort, the measurements that had a significant net change were the C2-C6 Cobb angle (-8.96; p<0.0001; d=1.09) and C7s (6.76; p=0.0003; d=0.92). There were no significant changes in the cSVA, C1-C2 angle, and the C7-CL from the preoperative visit to the last documented follow-up. In the lordotic cohort, the only net change that was significant at the last documented follow-up was the cSVA (5.23; p=0.041; d=0.53). There was no significant net change in C1-C2 angle, C2-C6 angle, C7s, and C7-CL.

Comparison of the difference in each measurement between the preoperative and the last documented follow-up between the cohorts is also given in Table 5. At the last follow-up, the only significant net change was in the C2-C6 Cobb angle (p=0.001; d=0.88). There were no significant between-cohort differences in cSVA, C1-C2 angle, C7s, or C7s-CL.

## Discussion

The sagittal alignment of the cervical spine is influenced by the adjacent spinal alignment to maintain global posture [26]. Degeneration of the cervical spine, as seen in degenerative disc disease, may cause kyphosis of the cervical spine and associated cervical radiculopathy requiring ACDF for symptomatic improvement [27]. Pre-ACDF sagittal balance was studied to determine the impact of ACDF on the restoration of cervical sagittal balance and overall sagittal balance. Post-ACDF, there was a significant restoration of CL in preoperative kyphotic patients, as shown by the significant change in the C2-C6 Cobb angle, resulting in a negative angle and the restoration of CL. The continued maintenance of reversed kyphosis over time was also supported by the strong d value, indicating a large effect size of the findings. The preoperative kyphotic cohort had a significant increase in CL at the first postoperative visit. The overall significant net increase in CL demonstrated the effectiveness of ACDF in the radiographic alignment over time, and the findings continued to demonstrate a strong effect size. CL was significantly higher in the lordotic cohort than in the kyphotic cohort at the first follow-up, a result expected due to the preexisting lordosis in the lordotic cohort. However, the small effect size of the comparison decreased the robustness of the strong comparative conclusions.

At the last documented follow-up, the C2-C6 Cobb angle did not vary significantly between cohorts, with a medium effect, showing that the lack of difference between the two is more substantial than the significant difference seen at the first follow-up. This finding can support the idea of ACDF maintaining CL over time, even in varying degrees of preoperative cervical sagittal alignment. The absolute value of the negative Cobb angle indicates the degree of CL, and the normal value of CL in asymptomatic volunteers has been reported to be 4.89±12º [28]. Both groups had values similar to this cited reference value. A study by Cheng et al. demonstrated the efficacy of the restoration of CL even at two years postoperatively in patients with a cervical extended range of motion (eROM), compared to those without eROM. The study also showed that both groups of patients had radiographic CL close to the normal CL level [22]. The authors additionally discussed the utility of applying the radiographic measure of loss of cervical lordosis (LCL) in assessing successful functional outcomes following cervical laminoplasty [22].

We studied the cSVA as one of the cervical spine measurements, as it is one of the most implemented measures for cervical sagittal balance [18]. Studies performed with asymptomatic volunteers have found a reference range of 16.8±11.2 mm for the standing cSVA [29]. Our findings showed that both the kyphotic and lordotic cohorts had a significant increase in cSVA, with the kyphotic cohort experiencing the increase at the first postoperative visit and the lordotic cohort experiencing a net significant increase at the last documented follow-up, with d values suggesting a medium level of robustness. Postoperative cSVA at the last documented follow-up was not significantly different between the cohorts, achieving a similar value of about 20 mm in both. These findings are consistent with studies that also found an increase in postoperative cSVA following surgery, which stabilized over time [30]. Gillis et al., in their retrospective study of patients undergoing ACDF, observed that cSVA increased by 9% in the first six weeks following ACDF, but decreased by 5% after 12 months [30]. Our study demonstrated a stabilization of cSVA at the long-term follow-up, with both groups having similar values; the low d value does not indicate a strong effect size when comparing cSVA between the two groups, and thus can further support the similarity of cSVA between both cohorts at the last follow-up. A cadaver study explained the biomechanical effects of how an increase in cSVA was related to the postoperative increase in the biomechanical loads of levels adjacent to the ACDF site [31]. A cSVA greater than 40 mm has been the measure commonly cited as a cause for clinical concern, due its association with quality of life [18]. Our findings noted postoperative changes in cSVA following ACDF, and these are consistent with the idea that ACDF can lead to biomechanical changes of the spine, which in time, can use compensatory mechanisms to achieve a positive correction of the sagittal balance [32].

The cSVA and the TS, in tandem, can provide a useful measure to quantify subaxial CL to help maintain the cranial center-of-gravity and horizontal gaze [33,34]. A study by Park et al. found that an increase in TS is associated with an increase in the Cobb angle and a decrease in the cSVA [34]. Since C7s has been an acceptable surrogate for TS [16], studying changes in C7s can be useful in indicating whether the cervical spine is kyphotic or lordotic [35]. Le Huec et al. performed a prospective study in asymptomatic volunteers to determine reference ranges of cervical spine measurements [28]. They found that the median C7s is 20º and determined that those with C7s greater than 20º have a lordotic cervical spine, whereas those with C7s less than 20º have a neutral or kyphotic cervical spine [28,35]. Our findings were consistent with the literature and studied reference ranges, where the preoperative C7s in the kyphotic and lordotic cohorts were 15.9±6.2º and 23.5±6.0º , respectively (p<0.0001). Postoperatively, C7s significantly increased in both cohorts at the first follow up. In terms of robustness, these findings were medium to strong. While these values were significantly increased from the preoperative C7s, they were well above the 20º reference value for C7s [36]. However, at the last documented follow-up visit, the C7s decreased in both groups to reach an average of 23.8±15.0º in the lordotic group and 22.7±8.3º in the kyphotic group. The lordotic group also had minimal change in C7s from the preoperative visit, and the statistical effect of the change was minimal. Between the preoperative and the last documented postoperative measurements, the overall C7s increase remained statistically significant in the kyphotic group, whereas the overall change was not significant in the lordotic group. Our findings support the literature that the 20º reference value for C7s is a useful indicator of cervical sagittal alignment, and that ACDF at our institution has been effective in restoring CL to normal values.

The atlantoaxial C1-C2 angle has also been studied as a useful indicator of cervical imbalance. Nuñez-Pereira et al. found that the C1-C2 angle has been negatively correlated with CL [37]. Yoshimoto et al. reported that surgeries at the C1-C2 level, specifically fixation in the hyperlordotic position, have been associated with inducing a compensatory subaxial cervical kyphosis. They also noted that the C1-C2 angle, being the lordotic fixation angle, correlates with the C2-C7 Cobb angle [38]. Ultimately, throughout our study, comparisons in C1-C2 angle did not yield any statistical significance between both cohorts, as well as any changes over time both immediately after surgery and over follow-up. Additionally, no robust conclusions could be made, as d remained <0.3 across all comparisons assessing C1-C2. Le Huec et al., in their study looking at asymptomatic volunteers with the purpose of establishing reference ranges, noted that the C1-C2 angle remained constant, even with differences in the C7 slope [28]. This study also supports our findings of the C1-C2 angle measuring 28-30º in both cohorts, consistent with the reported reference mean C1-C2 angle of 29º in asymptomatic patients [28]. The C1-C2 angle remaining constant, despite significantly different C2-C6 Cobb angles, is suggestive of how pathologies affecting cervical alignment result in compensatory mechanisms that are not limited to the cervical spine alone. Cervical imbalance can affect all aspects of the spine [28].

Cervical alignment plays a role both in the structural integrity of the cervical spine and global spine-pelvic alignment through compensatory mechanisms, because a deviation in the normal CL can affect the sagittal alignment of the thoracic spine and the alignment of the lumbar and sacral spine too [36,39]. Roussouly et al. reported that associations between the lower cervical levels and the sacral endplate may be able to serve as an anatomic constant and a marker of spinal balance in asymptomatic individuals and could therefore be used as a reference for patients with radiographic deformity with the formula 99º - 0.1 (sacral slope) [40]. The significant change in the Cobb angle and C7s in the preoperative kyphotic group, and the lack of significant changes in the lordotic group is consistent with the idea that ACDF restores CL in patients with preoperative kyphosis and maintains CL in patients with preoperative lordosis. Also, these findings, in conjunction with the C1-C2 angle being constant in both cohorts throughout the study, are indicative of how ACDF does not disrupt the maintenance of global sagittal alignment, regardless of preoperative cervical alignment, both in the short- and long term.

C7-CL was significantly higher in the kyphotic cohort compared to the lordotic cohort preoperatively, and this is consistent with the literature showing that a greater C7-CL is associated with a higher degree of cervical malalignment [41]. However, there were no significant differences in the postoperative C7-CL measurements between the cohorts, and over time. The measurements were similar in both cohorts postoperatively, in spite of the preoperative measurement being significantly higher in the kyphotic cohort than the lordotic cohort. This supports the role of ACDF in restoring cervical alignment from kyphosis. This is also evident in the long term, indicating that structural outcomes following ACDF can be sustained over the long-term. Moreover, it also suggests that ACDF does not play a role in exacerbating damage to the alignment over time, regardless of preoperative cervical spine curvature.

Limitations

This study is limited by its retrospective nature, as patients could not be screened before undergoing ACDF. Additionally, follow-up intervals were not significantly different between groups, but an exact consistent interval was not established for patients, which may skew results and the conclusions generated for long-term radiographic outcomes. Additionally, in the radiographic assessment, the C2-C6 Cobb angle, a validated surrogate for the C2-C7 angle, was used as a measure of CL; however, not including C7 in CL measurements may have introduced bias in the results.

Additionally, the study did not control for the surgical technique and surgeon performing the surgery, which may introduce bias in the results. The study's focus on radiographic measurements, without patient-reported outcomes like pain, quality of life, or functional status, limits its ability to link radiographic results to clinical satisfaction or quality of life. Additionally, the need for imaging at least two years post-surgery may reduce the study's external validity, as these patients likely had ongoing symptoms prompting the imaging.

## Conclusions

Our study on the impact of preoperative cervical spine curvature on ACDF outcomes shows that both lordotic and kyphotic cohorts improved in lordosis post-surgery, nearing normal CL levels. The kyphotic cohort showed a greater improvement in the C2-C6 Cobb angle and the C7 slope than the lordotic cohort. Both cohorts maintained these improvements after continued follow-up, confirming the effectiveness of ACDF in restoring CL. Future research should explore how preoperative kyphosis and lordosis can affect the patient's quality of life and clinical satisfaction outcomes after ACDF.
